# Palimpsests and urban pasts: The janus-faced nature of whitechapel

**DOI:** 10.1371/journal.pone.0251064

**Published:** 2021-09-09

**Authors:** Shlomit Flint Ashery, Nurit Stadler

**Affiliations:** 1 The Department of Geography and Environment, Bar Ilan University, Ramat Gan, Israel; 2 Department of Sociology and Anthropology, The Hebrew University, Jerusalem, Israel; Tel Aviv University, ISRAEL

## Abstract

This article examines how palimpsests in city spaces are mediated and negotiated by pedestrians’ individual everyday experiences. The literature on city spaces and palimpsests is rich; however, it has not examined the sharing and fusing of palimpsests into everyday life. To fill this lacuna, we explore how pedestrians mediate the physical path of the parcellations and the layers of meanings accrued over the years. We describe what we term the “Janus face of Whitechapel Road” that characterizes the multidimensional and ever-changing face of London as a world city. We look at the different traffic hinges distributed throughout the urban setting and track people as they encounter these historical and aesthetic landmarks. The experience of London’s palimpsests is an exemplar of this Janus’s face, governed by transitions, time, duality, and passages.

## Introduction

The past is a major protagonist in large modern cities. It is embedded in the city’s architecture, parks, urban planning, art, and aesthetics, and is considered a fundamental driver of city shape. The relationship between the physical public space as an object in terms of palimpsest and the experiences of layered signification has been attracting broad scholarly attention [1–5]. Yet, despite its potential impact on the neighbourhood, and the urban sphere as a whole, the everyday experiences are not part of the palimpsest literature. This research aims to address the conspicuous dearth of micro-resolution studies that identify how everyday experiences play a critical role in the palimpsest and accompanied by traces of different times in the complex urban setting. What is the role of the past in the actual everyday experience of city spaces? How are the different layers of past city palimpsests experienced by different urban agents? This article examines how palimpsests in city spaces are mediated and negotiated by pedestrians’ individual experiences. Based on theories derived from Georg Simmel, Walter Benjamin, Henri Lefebvre, Latour and Michel de Certeau, the ways in which pedestrians sense palimpsests is examined to better understand the phase-in of everyday urban experience.

In line with de Lucio’s statement [6] that any definition of public spaces should include “a vision of public spaces as psychical places where people can participate in public life and where people’s claims can be voiced”, Ortiz et al. [7] defined public spaces as "places of interrelation, social encounter and exchange, where individuals and groups with various interests converge". It is now common to assume that planning and design have a fundamental role in the democratization of the use of urban public spaces and in the fight against social exclusion [8]. Synergetic aspects of subjective experience and action of the individuals who use the space in their daily life together with the objective aspects of planning [9–11] become a central element in the construction of public space and the public sphere. We focus on the ways pedestrians mediate the physical path of the parcellations and the layers of meanings accrued over the years. These palimpsest ideas were about all possible experiences of layered signification, about traces of old realities shimmering through in the text, in space, in all forms of communication. This enables us to look at the agglomeration of old and new layers of the city, and how the addition of new layers are re-assembled in an order that allows for multiple interpretations by different agents. The impact of city palimpsests on everyday experience can contribute to city planning and the future design of urban spaces. We fuse a theory of city palimpsests with the notion of the flaneur–the stroller or wanderer–who, by virtue of class and occupation, is free to observe and experience the streets of the modern city.

This case study focuses on the city of London and in particular on Whitechapel Road, which has a long tradition of hosting different sites that were needed by London but which for various reasons always existed on the fringes of mainstream society. Using space as an object in terms of palimpsest allows us to show traces of different times in the complex setting of a current important and active area that has deep ancient roots. The Janus-faced nature of Whitechapel Rd provides a good example of a traditional closely knit ethnic enclave in the liberal, open-minded and creative culture of London. Whitechapel Rd is inhabited by different users from different ethnic backgrounds who have adapted the space to fit their needs over generations whose histories are part of the fabric of the urban system. Whitechapel Rd is one of many axes that connect the inner Roman city to its arteries This west-east axis extends beyond London’s collective identity as a world city towards the East End, an ethnic enclave undergoing an accelerated gentrification process.

Starting with a theoretical framework that outlines the effects of the urban Palimpsest on the experience of public space and the public sphere of London, in the next section we present the research methods. The following section examines the socio-spatial roles of urban palimpsest, provides analytical measurements of the urban palimpsest points and assess the influence of the past in the actual everyday experience of city spaces along Whitechapel Rd. The last section concludes the paper, assessing the capacity of a given public sphere for adaptation—it creates a new public sphere, embedded with contrasts and tensions, fragile but highly flexible. A Janus-faced sphere, that from one hand provides traditional roles, and from other a fertile environment for social and economic prosperity.

### The theory of urban palimpsest

The word palimpsest is derived from ancient Greece *palímpsēstos*, "again scraped". It typically refers to a papyrus or parchment manuscript that has been reused (scraped) and rewritten on more than once, such that some of the previous markings remain visible. This term is employed in textual studies to describe the way different layers of writing can be detected on scrolls. Manuscripts were re-used for a variety of reasons. Sometimes papyrus or parchment was hard to find or expensive. At other times, texts were ’scraped’ to remove mistakes or because the original text was considered heretical. In a palimpsest, the older scraped-off layers are never completely destroyed before a new layer of text is added. In addition, the new layer may be unrelated to the previous layers. The slate is wiped but not clean, and the new text does not appear on a blank slate.

In colloquial usage, the term *palimpsest* is used in archaeology, architecture, geography and history to designate an object made or worked upon for one purpose and later reused for another. For this reason, the term palimpsest can refer to ruins, structures, remains, and/or foundations on which new buildings, arts, roads, or centres are constructed. Sometimes, the ancient is taken into account, but in others it is effaced and designed anew.

In scientific as well as cultural domains, the term palimpsest refers to the overlaying or superimposition of knowledge [12–14]. Eco [15]’s discussions on "the role of the reader" shed light on the palimpsest concept as an interplay between the object and its interpreter and its interpretation. In this view, It? depends on the object that can be interpreted, the interpreter with her/his role, perspective, knowledge and expertise and experience in the urban sphere, and a matrix of possible interpretations/possible constructions of the palimpsest. There is some unity in possible interpretations, even if they are opposed; yet neither one is correct. This inherently fragmented form and self-awareness with regard to the novel’s own effects suggests points of comparison with postmodern, metafictional works such as Italo Calvino’s If on a winter’s night a traveller [16] or Paul Auster’s New York Trilogy [17], which, through their systematic undermining of realist conventions, draw attention to their own textuality; Thomas Pynchon’s Gravity’s Rainbow [18] and Umberto Eco’s Foucault’s Pendulum [19], in which Lyotard’s ‘grand narratives’ disappear only to return in the ghostly, parodic form of the conspiracy theory or the secret history, are also apposite references. Therefore, the ways in which people feel this history or relate to it depend on subjective cultural aspects which also affect their palimpsest’s experience.

In urban studies, the term denotes an object in space that contains diverse layers that appear beneath the surface [20,21]. This is commonplace in iconic cities that have been inhabited, developed and built up over the centuries [22]. These various layers of city spaces can be temporal, spatial, and imaginative or invented, and their discoveries are emblematic of the continual transformations and embodiments of the palimpsest process in urban settings where each layer tells a story [23,24]. In geological research, palimpsests trace how people first settled, lived their daily lives and built institutions and homes. Similarly, aesthetic or religious trends can transform the shape of the city and turn it into enclaves defined by religious institutions, theology or forms of the imagination [25,26]. In urban planning, the concept of palimpsest is used to define the stages of construction of architectural monuments and the evolution of urban morphology [27], achieved either by preserving locations and monuments to the point of ‘freezing’ the landscape or by promoting the adaptive reuse of spaces and buildings [28–30]. Bartolini [31] noted that when the traces of a palimpsest can be found on the most recent plans, the historical layers, layers of hidden knowledge, and existing layers emerging from new ideas and structures simultaneously form a superimposed chronological selection process. Iconic cities such as Rome, Jerusalem, Istanbul, Alexandria, Berlin, Moscow, and many others reflect this dynamic and shed light on the memories, the forgotten, old and ancient constructions and the remains of life in all its forms. This concept can also describe cases where historical layers are simultaneously visible.

### Palimpsests and the Janus experience of city spaces

In “The practice of Everyday Life” Michel de Certeau describes the creation of urban palimpsests [32]. De Certeau suggests that a broader view of a city can be gained from an overhead position such as from the top floor of a skyscraper. People moving through the city are unaware of the key role they are playing in rewriting the “urban text” of the city that appears from this vantage point as a new level of the historical palimpsest. The city is provisionally created as a patchwork quilt of individual viewpoints and opinions. "The created order is everywhere punched and torn open by ellipses, drifts, and leaks of meaning: it is a sieve-order." De Certeau discusses the significance of individual “pedestrian speech acts” (p.114); i.e., the steps and walking patterns that are spatially ‘enacted’ and ‘lived out’ the space of the city (p.114). These may form a palimpsest itself on a microscopic level by constantly superimposing new routes and “pedestrian enunciations” (p.116) on old ones. To see them, it is just as necessary to zoom out and perceive the grander “symbolic order of the unconscious” (p.117) that they form.

The term palimpsest is used in urban literature and in works on geography primarily by Italian researchers as an elementary, descriptive mapping strategy for dealing with the space and forms of territories [33–35]. In urban geography studies, the term is used to account for urban spatial and temporal development while alluding to "the meeting/clash between different times, endless modifications and transformations" [36]. André Corboz [37] proposed to think about territory as a palimpsest (le territoire comme palimpseste), a canvas conveying a three-dimensional matrix of signs, which forms a contextual bridge between the parchment document and the city and/or territory [38]. Parchment, which has handed down the scriptures since the Middle Ages, was a precious material that was reused, abraded, sometimes inverted and rewritten several times, while never losing its ‘stratification’, the traces of which remained legible. The city and its territory are like a parchment, transformed by the evolutionary history of the site, and endowed over the centuries with different meanings in relation to the societies that modify them. Corboz went beyond the modernist paradigm of tabula rasa and the ‘reclamation of the past’ as pursued by post-modern theoreticians [39–41]. He theorized territory as a palette upon which society can write; i.e., trace, erase, and ultimately re-trace new chapters of its urban transformation, without however erasing its signs, marks, and voids, both natural and anthropological [42,43].

The concept of urban palimpsest tends to draw on a small number of components that includes buildings, the image of the city (the physical features of the urban system), stages of spatial dynamics of the city during the reference period, and the territorial development factors and elements that mutually condition the current urban configuration and its dynamics. Huyssen [44], however, also considered urban expanses, monuments, architecture, and sculptures to be spatial palimpsests. Following Huyssen’s footsteps‏ to enhance the urban planning paradigm, we describe the built environment as spatial palimpsests that fuse symbols and architecture with traces of the past, thus creating historical memories of these expanses. This perspective contributes to a better understanding of urban landscapes as lived spaces that shape collective imaginaries. Following these notions, Whitechapel can be interpreted as an urban palimpsest, a powerful canonical marker of spaces that bear traces of the past, and memories of these contested sites at earlier points in their history. Put differently; they are heterotopias or hegemonic outposts that shape collective imaginaries, such as the ever-changing reflections of Janus’ dual experiences.

### Overwriting the cities: The palimpsest of the roman Londinium

**Fig 1:**
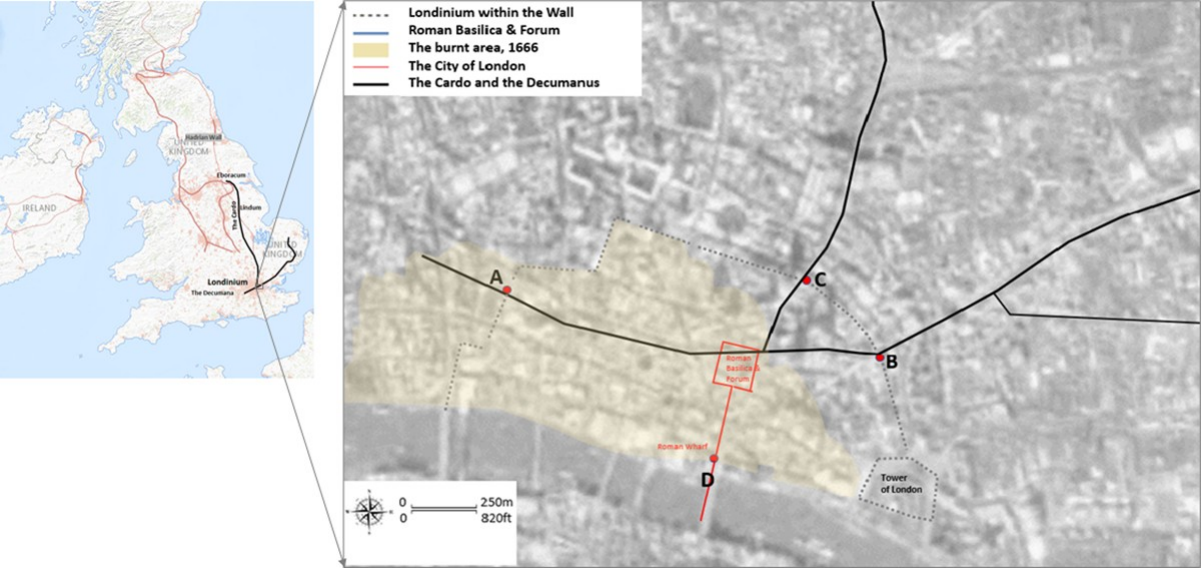
Patterns of the Roman occupation in today’s London: (a) The Roman Occupation of Britain and the location of significant settlements and roads during this period; (b) The Roman Londinium and the Burnt area of medieval City of London 1666 (inside the old Roman city wall) superimposed on a modern Google map to describe the connectivity of our case study. Note that since the Roman times the river has been embanked and so the wall’s line on later maps is seen to be slightly inland (https://landsat.visibleearth.nasa.gov/view.php?id=78669 and https://apps.nationalmap.gov/viewer/).

The city of London can be viewed from the standpoint of the theory of palimpsest and as an example of the Janus face to reveal its urban, cultural and narrative imaginaries. The settlement of Londinium was built on what is now the City of London around 43AD but also covered a small part of the Southwark Thames waterfront (Fig 1A and 1B). At its height, by the mid-second century, Londinium had grown to perhaps 60,000 people and was considered to be the capital of Roman Britain [45]. Along with Londinium’s forum and basilica, Hadrian’s Wall and the defensive wall around the landward side of the city, the road network was one of the largest construction projects carried out in Roman Britain (Fig 1A). Seven routes ran to or from Londinium, most of which were constructed around the time of the city’s founding [46]. By the second half of the second century, Londinium appears to have shrunk, and with the collapse of Roman rule in the early fifth century, London ceased to be a capital. From around 500, Lundenwic, an Anglo-Saxon settlement, developed slightly west of the Roman Londinium and by about 680, the city had regrown into a major port. From the original core of Roman Londinium, the city has sprawled in every direction, and a constellation of hamlets, market towns, suburbs and industrial areas have been displaced with each period of growth. Many different characteristics, topographies, geologies, watercourses and transport arteries, as well as social, cultural and economic drivers have merged and overlapped with one another and have had a profound influence on the anatomy of today’s London.

London’s layered history, including its varied neighbourhoods and its architecture, makes it an excellent example of an urban palimpsest that has erased and preserved traces of layered cultural histories and peoples over time [46,47]. Although it has become one of the world’s most important global cities, the City of London, London’s ancient core, has retained Londinium’s boundaries of just 1.12 square miles (see border in red, Fig 1B). Also known as the Square Mile, this is the current heart of the financial district, which is administrated by a separate body than the rest of Greater London which encircles it. After its reconstruction around AD 60, the area preserved its close urban grain whose key determinant is its web of narrow streets. Apart from the riverside wall, which was probably knocked down by the Saxons, the rest of the Roman Wall was still in place until 1760 when most of it was demolished. The original trace of the wall accounts for some of the street alignments around the City today. Today, as before, the historic street pattern has endured, accommodating high densities and a range of offices for commercial and civic uses.

Within this grid pattern of the City of London, most of the Roman gravel roads can only be identified today if they were repeatedly resurfaced or if the spans of gravel can be traced across several sites based the area’s geology [48]. Many of the stone structures have left foundations, but these were often dismantled for their stones and used to build new constructions during the Middle Ages and the early modern period [49]. Similar to other ancient Roman cities, the main streets of Londinium were the Cardo, the north-south road, and the Decumanus, the east-west road. The Forum was typically located close to the intersection of these roads. The Cardo of Londinium was interrupted by the Forum north of its intersection with the Decumanus. Usually, 9–10 m wide, the Cardo entered the walls of the city at what is now Bishopsgate (point C, Fig 1B), which was also the start of Ermine Street, the main Roman road that ran north to Lindum (Lincoln) and Eboracum (York). At its other end, point D indicates the intersection of the Cardo with the southernmost stretch of the southern wall. This was the Dourgate (today Dowgate Hill) where water flowed from the Wall Brook. The name derives from the word dour (or dwr), the Celtic term for water. Point A on the western end of the Decumanus, also known as the Great Road (Fig 1B), indicates the Roman New Gate close to the medieval St Paul’s Cathedral. Since it is located at the highest point in the area, the Elizabethan antiquarian William Camden argued that a Roman temple to the goddess Diana had been erected on the site now occupied by the cathedral. However, there was no trace of a temple during the works to build the new cathedral after the Great Fire, and the location of Londinium’s original cathedral remains unknown. Point B indicates the Eald or Ald-gate, the Roman burial way. From this point, the Decumanus ran northeast across the Old Ford to Camulodunum (Colchester—already a British tribal centre) and then northeast along Pye Road to the Venta Icenorum (Caistor St Edmund). The urban line between the Roman Eald Gate (point B) along Whitechapel Road to the ethnic enclave of Whitechapel market [50,51] is the focal point of this study.

## Method

Any exploration of the design process of city spaces must involve a careful observation of the historical layers to better understand how they were erected, eradicated, preserved or developed over time [52,53]. The palimpsest provides a lens to investigate these layers separately and sheds light on the ways in which new ones were added. These layers can be reassembled in an order that enables multiple interpretations. The palimpsest strategy in this context involves incorporating site-inherent values into a new design or system [54,55]. The research methods implemented here combine quantitative and qualitative data analysis of overlapping map analysis and in-depth interviews, combined with a morphological analysis. To better trace the impact of the urban palimpsest, all the points identified were classified as either palimpsest (P, in blue) or newer points (N, in red).

### Overlapping map analysis

To trace changes in urban patterns, to identify what remains, what has been transformed or demolished, and to reveal trends, we analysed the physical path of the parcellations through a spatial analysis of overlapping maps. This data mapping was carried out by scanning, digitizing and converting maps (below) using several GIS applications programs:

Roman Londinium c. 225 CE (www.themaparchive.com)

The map of London in 1572 (Braun and Hogenberg, Civitates Orbis Terrarum)

After the Great Fire of London 1666 (The Yale Map Collection: European Cities)

Plan des villes de Londres et de Westminster et de leurs faubourgs avec le Bourg de Southwark, 1700 (Nicolas de Fer)

A Plan of The City of London, 1720 (Westminster and Borough of Southwark with the new additional Buildings. Annoxxxx.)

The map of London in 1859 (John Snow ’s map, prepared by Ralph R. Frerichs Department of Epidemiology at the UCLA School of Public Health)

London Poverty in 1898–99 (Charles Booth Poverty Map & Modern Map, London School of Economics) and google.com/maps/.

The comparison between the maps allowed us to identify important nodes on the axis of urban development of the area from the Roman city to the east while referring to the local topography and morphology and distinguish between places that were preserved and places that changed over the years.

### In-depth interviews

To better understand the layers of the city spaces, uncovered by the overlapping maps analysis, and to decipher meanings and experiences of people walking in urban spaces, we conducted 103 one-hour in-depth interviews (63 females and 40 males). These interviews allowed an understanding of the nuances of voices manifested through the layers of the city. We have interviewed different age groups (all over 18) and most of them were living in the neighbourhood. The interviews were conducted with shop and stall owners and workers, businesspeople and visitors who came to the area either on foot, on the tube or by bus. The interviews took place at the meeting points, were audio-recorded and written notes were taken during or after the interview.

The interviews adhered to an interview guide, which provided a list of open-ended questions to shed light on the ways palimpsests play out in the urban fabric for passers-by of various types and experiences of different people. The interview data were analysed using open-coded thematic analysis. Over 90 per cent of the interviews (94 interviews) referred to the historical space in statements such as "the area is beautiful because it is ancient" or "back then they knew how to plan cities". These insights are meaningless when constructing the different layers of meaning and explaining the unique experience of walking in the city. We, therefore, carefully selected the quoted interviews to emphasize the local, vernacular perspectives and pedestrians’ individual everyday experiences of urban spaces. They indicate that this is a relatively busy area, with ethnic aspects and local cultures, that palimpsest idea is about possible experiences of layered signification, about traces of old realities shimmering through in forms of socio-spatial communication. All the participants’ names have been changed to pseudonyms. The analysis of these interviews allowed us to distinguish between gathering points that create, and do not create, gathering spaces. P1 to P5 appear in London maps before the fire and since 1572 (Braun and Hogenberg, Civitates Orbis Terrarum) as landmarks (e.g., Whitechapel church) onwards. N1 to N2 appear as gathering points only from the end of the 19th century (e.g., with the Aldgate stations’ opening). Observations in these areas enable us to define agreed meeting points, where at least three meetings are held simultaneously, and meetings last over five minutes (including waiting time) for two or more individuals. These gathering points differ in terms of the time of day and uses frequency. There are places where street benches or wind-sheltered areas allow longer stay and others populated mainly at certain hours, for example, at the beginning and end of the working day. Others are especially populated on weekends. These places attract different segments of the population. The overlapping map analysis and the interviews enable us to recognise further urban palimpsests anchored in ancient historical sites and locally acknowledged settings.

### Morphological analysis: Isovists

We use the behaviourally and perceptually oriented methodology of Isovist to better understand the users’ walking patterns and space accessible for visual and physical contact. The concept of isovist was introduced by Tandy [56] and is widely used by the Space Syntax community [57]. Every point in physical space has an isovist associated with it. An isovist is defined as the volume of space visible from a given point in space, together with a specification of the location of that point.

The Isovists.org platform for advanced spatial analysis of urban areas to construct a set of two-dimensional isovists in the horizontal section of Whitechapel Rd. We adopt Turner’s [58] differentiation between *Accessible Isovists*, which include all space seen and directly accessible from the origin point; and *Visible Isovists* which include all space seen but inaccessible from the origin point (such as space beyond a void). We use the isovist technique for each gathering point and recognise the movement area available for actual walking trajectories at each gathering point from the overall visually accessible space. The analysis of values in a sequence of viewpoints along Whitechapel Rd. is directly applicable to planning issues. For the other sampling methods, such a sequence would not have such a direct meaning for planning issues because adjacent viewpoints are not necessarily directly physically accessible. Views and quality of open space observed from different alternative locations are changing according to the morphological conditions of the surrounding as depicted by isovists, enable us to better explain how palimpsests in city spaces are mediated and negotiated by pedestrian experiences along Whitechapel Road.

## Results

### The socio-spatial roles of urban palimpsest

Whitechapel Road is a part of the historic Roman Road that goes from the Londinium Forum to Camulodunum (Colchester). As it phases in from the Roman Londinium towards the East End, the area gradually reveals its distinctive architecture resulting from therich history of the city. Based on observations and interviews, ten main gathering points where pedestrians congregate were defined.

**Fig 2:**
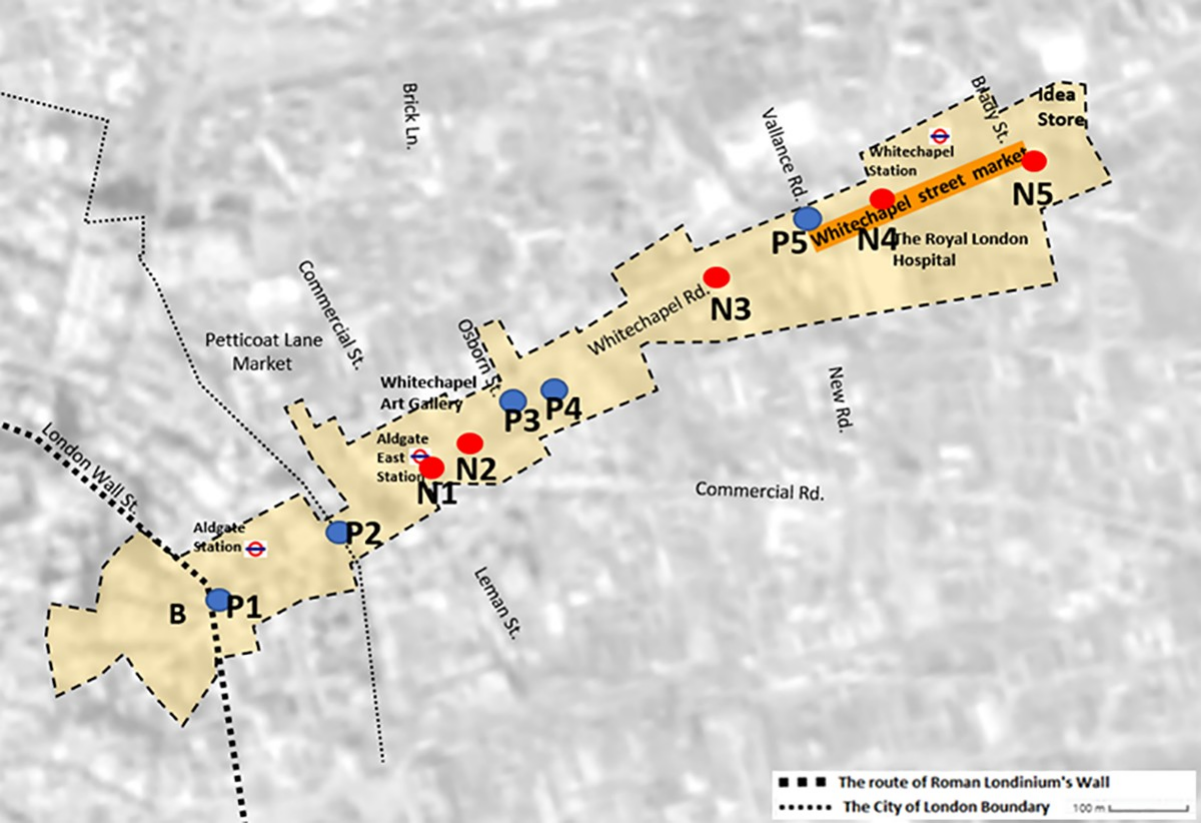
Whitechapel Rd: (1) Just outside the Roman Eald Gate and today’s Aldgate; (2) The border between the City of London and the London Borough of Tower Hamlets; (3) East Aldgate station; (4) Main junction of Commercial Rd and Whitechapel Rd; (5) Osborn St, the way to Brick Ln and Shoreditch; (6) Atlab Ali park; (7) The East London Mosque & London Muslim Centre; (8) The beginning of Whitechapel market; (9) Whitechapel train station for underground & overground; (10) Idea store and the end of Whitechapel market. Note that this figure presents the longitudinal axis of Whitechapel Rd. To highlight the gathering points within their surroundings, Fig 2 was limited to include only the entire buildings adjusted to the street-front or those connected through a common yard or function. For example, the hospital has eight adjoining buildings, the area around Whitechapel tube station, East London mosque and the gallery (https://eoimages.gsfc.nasa.gov/images/imagerecords/78000/78669/london_etm_2002256_321_lrg.jpg).

Fig 2 depicts five of these main gathering points, labelled P1 to P5. It shows the connections between the points and indicates that new developments and ’memory-rich areas’ lie adjacent to each other. P1 is just outside the Roman Eald Gate, in today’s Aldgate. It is the starting point from which a ribbon of development spread along busy mercantile Whitechapel Road throughout the years. P2 is located on the boundary between the City of London and the London borough of Tower Hamlets. This area developed gradually from medieval times until the late 16th century and today has a large number of office buildings and several institutions. The meeting point between Whitechapel Rd and Osborn St, indicated by P3 is on the way to Brick Lane and the highly gentrified neighbourhood of Shoreditch. Several of the city’s best-known night clubs are located here. One was the scene of a nail bombing attack against ethnic minorities and gay people that injured 13 people in 1999. Atlab Ali park (P4), formerly known as St. Mary’s Park, is the site of the 14th-century white church from which the area of Whitechapel gets its name. The park was renamed in 1998 in memory of Altab Ali and other victims of the racist attacks. P5 is located at the meeting point between Whitechapel Rd and Vallance Rd and is the traditional starting point for the established street market of Whitechapel. These points are approximately 17 to 31m (56-102ft) above sea level and are described on the deed records as reserved for “non-residential use”. The estimated average property price in 2020 is approximately £3,224,236. The average commercial rateable value is £322,500, and the most common recorded types of businesses are offices and car parks.

A group of students from a local university gather to summarize their London Wall Walk. After the group disperses, Lea (F28, P1) explains the advantages of this gathering point:

C1: “We bring students here at least once every semester and this location is great for observing crosscutting relations and to do some basic mapping. When the Roman City Wall was built, a stone gate perhaps already spanned the Roman road linking Londinium with Camulodunum. We believe that the gate had twin entrances flanked by guard towers because the medieval gate also had a single entrance flanked by two large semi-circular towers. Outside the gate, a large cemetery developed to the south of the road.”

Six young businesspeople are sitting on two benches having their lunch. Janet (F22, P2), one of the four women, has not been outside the City of London:

C2: “We came for a few months of work experience in business with a substantial insurance brokerage component. We came from Manchester to work in this prime location, and since then I have been here with this group. We meet here for lunch—there’s a competition over these benches—I have never seen the benches unoccupied! My period is almost coming to its end, and I need to meet people and make connections during the lunch break. I haven’t found the time yet to walk around, but anyway, I have everything I need here.”

Uddin (31M, P4) of Bangladeshi ethnicity describes that people feel the local history and relate to it:

C3: “We are all of the same Chula (cooking pot), so everyone who is related to me through ’father to son’ is my family. The house is crowded so we spend most of our time in the park. In the winter the children draw on the frost with their fingers and when it gets warmer the families eat together. There are lines here on the paving, where the old church stood and even the youngest are already practising walking heel to toe. That’s until the evening hours when we see the rest of the family coming from the city. “

Bhavna (F53, P5) chats with two elderly women outside their family’s ladies clothing store near Vallance Rd. and explains the local set of rules that everyone accepts and obeys.

C4: “We (the older ladies) are here at the beginning of each week help when the groceries are delivered. From here we have a good view of the City, Vallance Rd., and the mosque and we can see who is coming without been seen. You won’t see any young Sylheti women here. We keep them inside the shops or stalls and within their families. But for us the situation is different. When we are here men will not come out, but when we leave, they will come out to talk for hours! But if I need a man in the shop I won’t come out when men are around, I’ll text my husband to come to the shop. We work and live here, in the market, and sell saris and the Salwar Kameez collection. It is a funny building, it used to be the ‘Red Lyon, Towns End’. It’s written inside. Now it is our shop but before it was a pub and we can still see the décor that was here in 1730 or so…"

The five "newer" gathering points are current meeting places that have undergone refurbishments, and whose scale and parcelling have changed significantly to accommodate the rise in traffic. N1 indicates the East Aldgate station that opened in 1884 at the intersection between Commercial St and Leman St, underneath Whitechapel Gallery (opened in 1901). N2 is located at the main junction of Commercial Rd and Whitechapel Rd. N3 indicates the East London Mosque and London Muslim Centre, located next to the former Whitechapel Bell Foundry company that dates back to 1570. N4 is located at the main entrance to Whitechapel station. N5 indicates the meeting point between Whitechapel Rd and Brady St. It is the location of Whitechapel’s main public library (idea store) and the end of the Whitechapel market. These points are approximately 14 to 25m (46-82ft) above sea level. While N1 and N4 are described on the deed records as "mixed residential and non-residential areas", they, as well as N2, N3 and N5 are now primarily non-residential. The estimated average residential property price in 2020 is approximately £566,924. The average commercial rateable value is £70,203, mostly for shops and offices.

Originally from the Highlands, Thomas (26M, N1) works at UCL. His brother had moved to France, and his room in Leman street was vacated. He moved here a few months ago.

C5: “The location is great, close to everything, and everyone can feel at home here. Brick Lane has signs in Bangladeshi, and there are buildings with inscriptions in Hebrew and the Huguenot. There are many ancient British heritage sites all around. I read this article about the archaeological digs at Boar’s Head playhouse, and I thought that although my belonging is less clear to others as a newcomer, I feel so belong to this area.”

Aashi (F21, N2) explains that at certain times of day, local males congregate at this point:

C6: “I have finished cleaning and I am waiting for my husband. It’s because I’m not comfortable being here because there are only men here at these hours, usually accompanied. This is a big junction complex with lots of traffic lights and employers pick up and drop off employees. Other men in the area are mainly from the local Somali and Pakistani communities. So after a day of work, we meet here and walk home down the main road.”

Azam (M36, N4) describes the entrance to Whitechapel station as a meeting place for men from the Bangladeshi community:

C7: “This is a good place for people watching because many of us (Bangladeshi men) sit together for *adda* [to chat] twice a day before crossing the road to the mosque and there are crowds of people going in and out of the station. The market is for the community, and sells clothes, jewellery, fruits and vegetables, so there’s no competition here (for selling watches). I have eight children. One of them is here, look! I need to move before the inspector comes. I can walk to the city; I am not taking the train.”

The road is 1 mile long from the Londinium Forum to the Whitechapel market, and the Whitechapel street market itself is 0.3 miles long. Along the way, the building blocks of Whitechapel’s complexity merge to reveal the agglomeration of old and new layers of the city. Occasional visitors, old-timers and new residents from different backgrounds all have a different level of influence on the built environment as they go about their daily lives (e.g. gathering), unaware of their profound involvement in the ongoing process of the city’s formation. Whereas the P points indicate the corners of the streets which are undergoing another incarnation with new users, the significant refurbishments of the N points suggest they are transiting to their future role as iconic points in the area. One travel guide describes this area as ’full of noise, colour and life…a palimpsest of culture’.

### Validation of the urban palimpsest points

**Fig 3:**
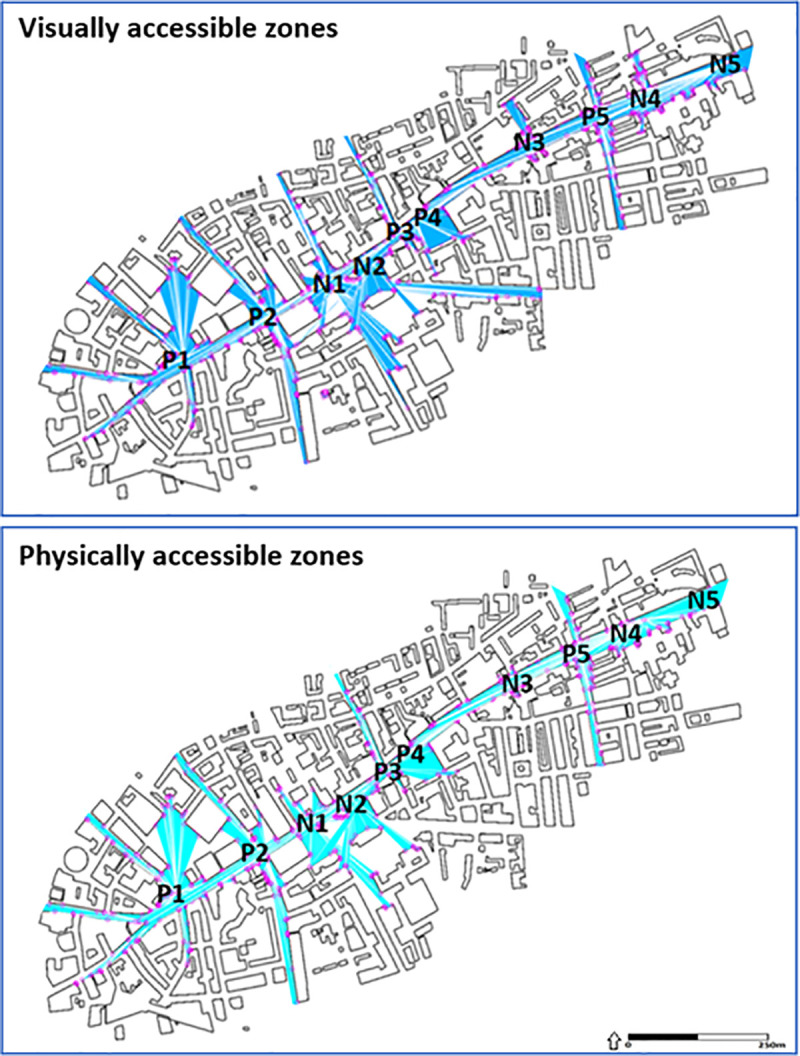
Isovists: (a) Visually accessible zones of each gathering point; (b) Physically accessible zones of each characteristic location. (The analysis was performed in Isovist.org software 2020).

Fig 3 shows that there is a differentiation in terms of accessibility between P and N points. P1 and P2 are in the westernmost part of the road, within and on the border of the City of London. The visually accessible zones of P1 and P2 have a view towards the City and along Whitechapel Rd up to P4. N1 and N2 are adjacent to each other, are located on a central junction that has undergone a significant parcellation and renovation. East Aldgate station was moved to its current location in around 1938. Due to the local topography, both have limited visually accessible zones that mainly encompass the immediate area. P3 and P4 are also adjacent to each other. In addition to the broad exposure to the main road (including P1 to N5), P3 has direct access to the gentrified Shoreditch area (through Osborn Ln) and P4, Atlab Ali park is a popular meeting place used by the business people working nearby, children from nearby kindergartens and schools, as well as for matchmaking. N3 indicates the location of the East London Mosque (construction began in 1982 on land left vacant after the bombing during WWII), which incorporates the London Muslim Centre (2002–2004) and the Maryam Centre (2011–2013). It is the most active Islamic institution in the UK, accommodating more than 7,000 worshippers for congregational prayers. Although we are unable to access the view from this location, it can be assumed that just as in 1982, the view is restricted by the curve in the road. P5, N4 and N5 are associated with the market. P5 and N5 are located at the ends of Whitechapel market. N4 is located in the centre, at the entrance to the bustling Whitechapel station, which also serves the hospital located opposite. P5, the traditional starting point of the Whitechapel market, has a view on the wide Vallance Rd (both directions) and towards P3.

N4 and N5 have open views towards the east but its views towards Greatorex St are limited. These views are even more curtailed during market hours because of the stalls. The comparison between the visually accessible zones of each gathering point reveals that the palimpsests ("P") points have more "views" than the "newer" ("N") gathering points, from which the panorama is more bounded. In addition, the locations of P points can be seen from other P points.

Ronan (M26, N5) sits outside the idea store and describes the benefits of N5 and P5:

C8:"I have been sleeping in the rough for a few years now. I know all the places here to hide from the rain and police and all the people around. People are fighting over space here. Behind the mosque, where the old synagogue used to be, there are few sheltered places where everyone wants to stay at night. The hospital also is a great place. If you turn right, you can see the place where I wash. I have everything here in the neighbourhood. To sleep and have some quiet space, I am here (N5) and the NGO people bring blankets and hot meals. To beg and see people I move on (to P5), but often there is already someone there."

These two invisible mechanisms point to the dual socio-spatial role of the local P points: (1) To link, by sight and accessibility, the centre with the urban fringes, the ancient Roman City and the modern City of London, and its current incarnations which are also located on the outskirts of the City; (2) To enhance the relationships between the centre-culture and the multi-identity alternative culture of the immigrants, who gain an additional perspective by observing from other P points. This suggests that in addition to their location, being observed and reflected from other P points reinforces their importance.

Itzhak (M83, P3) a former resident of Whitechapel, stands on Whitechapel Rd/Osborn St and recalls this large WW2 bomb site:

C9: “You can walk here from the city in a few minutes, so everyone wanted to live here despite the harsh conditions. This area was 30–40% Jewish back then, with Irish, Maltese and all sorts of people who also had a crazy council apartment… In 1958, my brother worked in a clothing factory on the upper floors of this corner building, which is now a branch of the Turkish restaurant Efes.”

An analysis of the qualities of the points makes it possible to associate the nature the N/P points to those of the agents, who add layers and reassemble interpretations while supporting the endless process of superimposition of spatiotemporal knowledge. In line with Bartolini’s (2014) description, the agents/users of a particular point reassess and form their identity while referring to the vertical layers of the past. In a natural-selection-like process, only some of the historical and hidden knowledge layers are exposed as traces of the palimpsest and merge within the contemporary urban setting. This process, however, is not necessarily superimposed chronologically. In Corboz’s theorization, the 3D palette enables individual agents/users to select, reproduce and edit their own layers while defining themselves through their activities. Here, extending notions developed by Huyssen (2003), the components of urban palimpsest are not the objects themselves (e.g., buildings), but rather the spatial interaction between the physical aspects of the urban system and their past, between themselves (as observed and reflected from other points) and with their users. This viewpoint allows individuals and groups to define themselves through their spatial interactions.

### Flânerie down whitechapel road

#### From West to East

The role of the past in the actual everyday experience of city spaces and how city palimpsests are experienced by agents perhaps best emerges by taking a pedestrian route from the city centre and/or back along Whitechapel Rd. The in-depth interviews and observations suggested that there are palimpsest relations with other images, one overlaying another.

**Fig 4:**
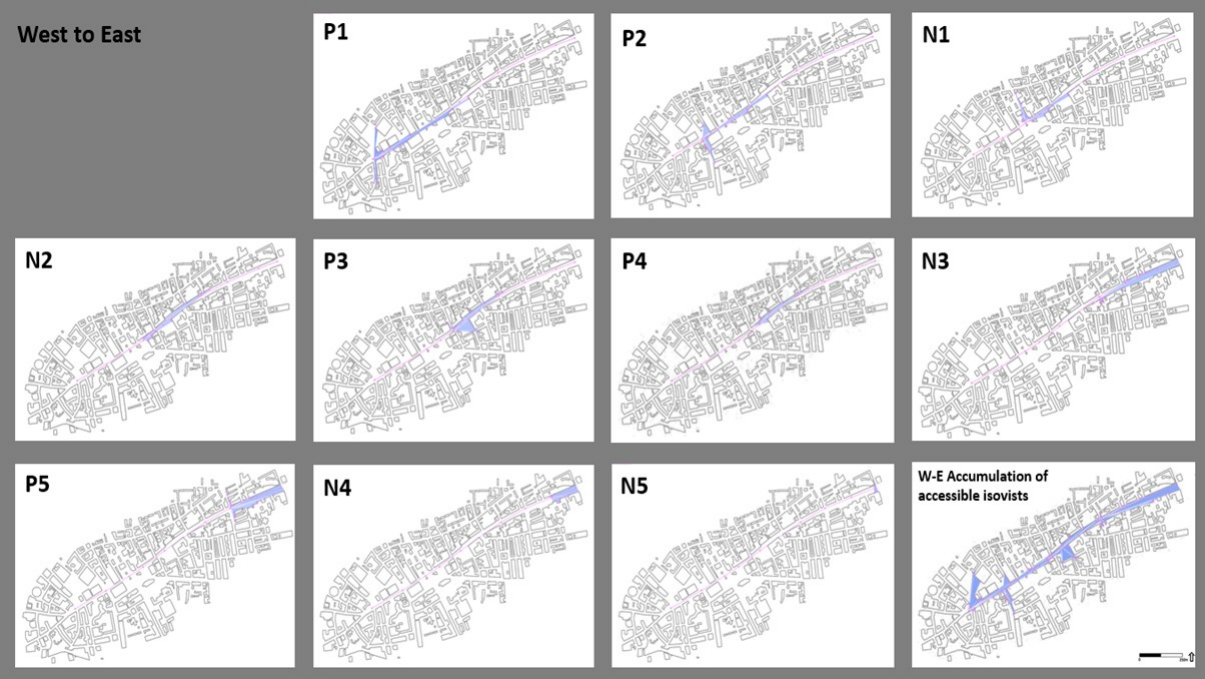
West to East: Accessible isovists by points and their accumulation.

Fig 4 reveals that P1 and P2 allow people who congregate by the Roman Eald Gate, in today’s Aldgate/municipal boundary, to have good access eastward. P1 is located where the Boar’s Head playhouse at Aldgate (1598 to around 1616) was recently excavated. P2 is the highest point in the area, and the points cover wide areas, with large perimeter extensions and good vistas eastward and up to P4. Properties near P1and P2 pay more than £68,000 and up to £88,000 (tax band D), the highest within this research area, and are an indication of the most attractive locations.

N1-2 are relatively low on all parameters, indicating a more limited view of the context. However, N1 has a view of Old Castle St, perpendicular to Whitechapel Rd. and faces north whereas both N1 and N2 look eastward towards P4. Properties near N1 pay more than £40,000 and up to £52,000 (tax band B), the lowest taxes in the research area. Properties near N2 (including N2-5, P3-5) pay more than £52,000 and up to £68,000 (tax band C), the average tax for Tower Hamlets.

P3 is on Whitechapel Rd/Osborn St and the nearby P4 is on the site where the first church was built in the mid-thirteenth century, dedicated to Mary de Matefelun and currently known as Atlab Ali park. These points are favourable in terms of their area and perimeters. The vista from Atlab Ali park towards the East is particularly vast (VP4, 119.54), and it must have been possible to see far into the distance from the church.

N3 is the lowest point on all parameters. An archaeological dig during groundworks at the East London Mosque only uncovered finds that date to the post-medieval period. P5 has the most significant parameters in this direction (AP5, 224.66; PP5, 252.09; VP5, 109.27). It comes as no surprise that at a nearby site to this prime location (85 Stepney Way, next to the Royal London Hospital built in the 1750s) archaeologists have recently found remains of The Red Lion, the first permanent home for acting troupes and staged plays by Shakespeare (from around 1567). N4 and N5 have similar low parameters although N5 is located at the lowest point in the research area, approximately 14m (46ft) above sea level, and therefore easily controlled.

Adam Single, an archaeologist associated with the Greater London Archaeology Advisory Service (GLAAS) claims that the relationship between the religious and cultural sites in this area (N3 to N5) resulted from geopolitical opportunities and constraints:

C10: “… the two biggest factors in the choice of location for the Red Lion are the liminal location on an early modern period political boundary and also the legacy of a long history of earlier occupation, which was encouraged by the local physical geography. … The location on a political boundary is shared by other playhouses of the period, which seem to have been sited far enough away from the City fathers to avoid interference and oversight of an activity that was often deemed undesirable but which still relied on being close enough to population centres to draw a good paying crowd. There was a great fear of plague at the time too and the risk of contagion has been raised as a factor in situating the Red Lion a full mile away from London. Possibly the Red Lion was unsuccessful as a commercial venture because it was slightly too far away, in fact.”

The walk from the Roman Eald Gate towards the east preserves the accumulated linear views of the route, at least up to P5. In line with de Certeau’s "pedestrian speech acts", the footsteps and walking movements that spatially ’act’ and ’live out’ Whitechapel Rd may form a palimpsest itself on a microscopic level by constantly superimposing new pathways and "pedestrian enunciations" on older ones. Thus, it is just as necessary to zoom out and perceive the grander "symbolic order of the unconscious" that they form. The patterns the flaneur perceives as s/he walks down the road are simultaneously visible layers of hidden knowledge, and existing layers that are a combination of new ideas and structures. They are not the truth (they do not reflect the Roman’s desire for power and control over the vacant area outside the walls) but ’fiction’, arrived at through the intoxicating effects of the walk and the urban environment itself. However, the vista from Altab Ali Park toward the brick social housing projects of the East End reveals the current truth about some of the most deprived areas in Britain. The constant gentrification and embodiments of the never-ending palimpsest process show the limits of flânerie for understanding the changing face of London.

#### From East to West

**Fig 5:**
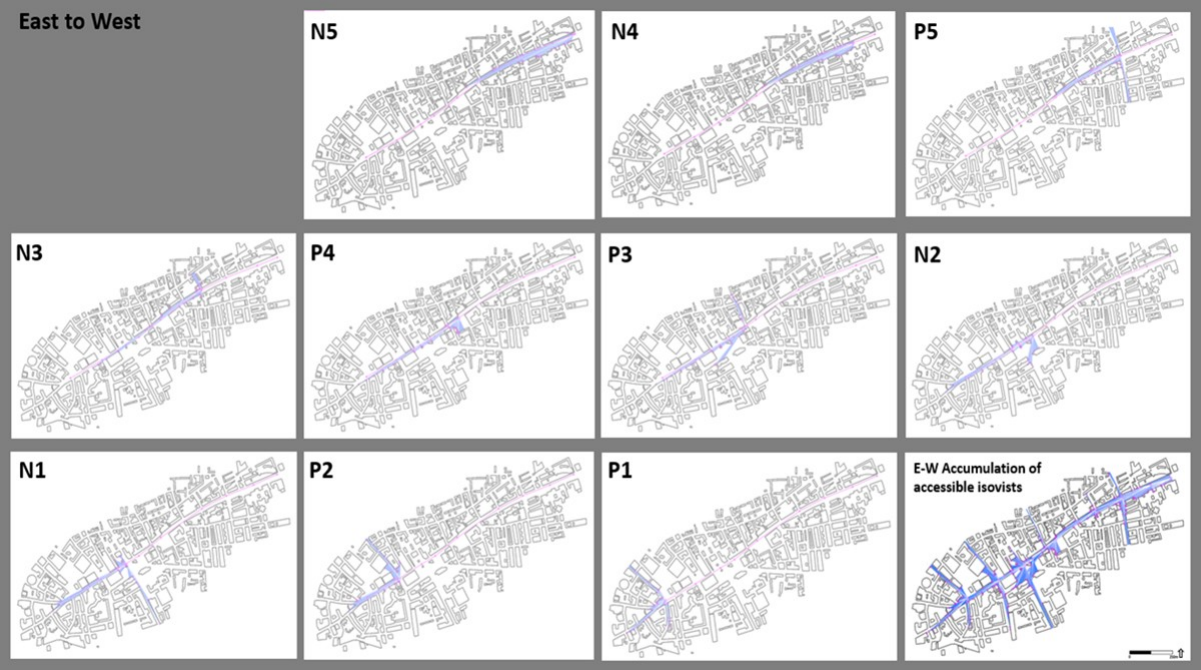
East to West: Accessible isovists by points and their accumulation.

Walking along Whitechapel Rd from the end of Whitechapel market (N5) towards the West in Fig 5 reveals that N4-5 maintain their low area and perimeters, as in the other direction, while having a vista towards N3. Adam Single describes this terrain as follows:

It’s possible that the course of a now hidden stream, called The Black Ditch in historic times, was used to define the parish boundary. We know from the archaeological work that this site at Mile End/Whitechapel was attractive to occupation more than 2,000 years ago as we found a middle iron age settlement there. I expect that the water source from the Black Ditch and the dry location north of what would have been marshland close to the Thames made it a decent enough spot to live and farm.

Strikingly, from P5 onwards, and in addition to their vistas facing west, all the locations have access to additional views towards the south, north or both regardless of their parameters. Fragments of the big picture slowly reveal the interpenetration of public space through our flâneur’s experience. Views from P5 to the building blocks with shops along Vallance Rd. provide a view of some parts of Whitechapel that are populated mainly by the UK’s youngest and fastest-growing minorities that have continued to experience considerable social and economic disadvantages. From the same point, the view towards New Road reflects the structural and social changes caused by slum clearance in the 1970s and the war-time destruction replaced by council accommodations. The former New Road Synagogue, the Indian restaurant, the old clothing factories maintain the long history of Whitechapel as an absorption neighbourhood, attracting successive waves of migrants each of whom has added a new dimension to the culture and history of the area.

Views toward Davenant St. from N3 continues this story with a clothier’s warehouse and a denim factory (1850–1899), which was only recently (2008) converted into offices and flats. Most newcomers, most historical immigrants such as the Huguenot refugees, Irish Catholics and Jews worked in the sweatshops of the clothing industry where manual labourers were employed at very low wages for long hours under poor conditions. The adjacent sugarhouse building (1797) is the sole surviving remnant of a once widespread building type in Whitechapel.

Altab Ali Park (P4) with its inner view is symbolic of the Bengali community that settled in the area at the beginning of the 20^th^ century to work in industries related to construction and ship repair. From the mid-1970s many experienced racism, social deprivation and a high level of unemployment. The murder of Altab Ali, a leather factory worker in 1978, led to the mobilisation and politicisation of the community.

The view from P3 reveals the site of several of the city’s best-known night clubs which was the scene of the 1999 nail bombing attack described above. Today this popular meeting point is the way to Brick Ln and the highly gentrified neighbourhood of Shoreditch. With each flurry of snowflakes, new images form, yet the speed of the movement prevents them from ever being perceived in their entirety, only as moments of becoming and dissolution.

While approaching the financial centre of the City of London, the presence of economic regeneration, together with social policy activity, are felt more strongly. Views from N2 cover the areas of Commercial Rd/Whitechapel Rd/Commercial St and from N1, the area of Aldgate East station and Leman St. Within the City of London, P2 provides a good view of Middlesex St and P1 to both Jewry St and Dukes Pl. The much newer development here is growing rapidly, in tandem with the gentrification process and the construction of new offices and apartment buildings, as well as the remodelling and extensions of buildings. From P4 onwards, the view of the City is secure. However, the new high-rise buildings of the City of London, The Leadenhall Building, Deutsche Bank and The Gherkin block view as though they were the Roman wall.

The route from east to west emphasises vernacularity and locality, at least to the place where the high-rise buildings become visible. Voices along the way enact a motion from apparent coherence to dissolution. Walter Benjamin does not delineate the precise contours of the flâneur’s outlines but sets in motion the whirling fragments into which he can appear. Similarly, this short journey is composed of a series of fragmentary tales and images that reflect the contradictions in Whitechapel’s social base. The coherence that the subjectivity of the narrator appears to give to the journey—or the clear axis of the road—dissolves. As the walk continues, the tale of the neighbourhood jumps across eras, characters, and narrational perspectives. It then becomes dispersed through a series of related discourses. From these voices, a representation of Whitechapel that is alive to its contradictions appears, and which at all points refutes the possibility of closure of meaning into a single, linear reading of the City and its disparate spaces. There is a creation of socio-spatial nests with different qualities from the City. This did not happen in Roman times and was not planned but occurred as a gradual formation over hundreds of years in a particular place that defined itself differently from the City.

Naturally, the walk along Whitechapel Rd., both from west to east and vice-versa, is linear: the pedestrian walks the same distance and on the same route. However, as suggested by Viganò [59], there is a clash between the different experiences created by the local morphology, layers and meanings that have been added to the area over the years. We thus suggest the metaphor of the Janus face to represent the dual experiences of Whitechapel Rd. In line with Huyssen [60], we go beyond the limited components of the urban palimpsest concept to determine how the axis dichotomies are mediated and negotiated by pedestrians`experiences. The strength of this area lies in in its ability to produce a world city while also preserving history and in the production of a distinct vernacular culture.

The findings suggest that Whitechapel’s appeal emerges while walking. For the pedestrian, the overlay of today’s London is inseparable from its essence and the pedestrians themselves. Unaware, as de Certeau claims, of the critical role they play in writing the "urban text" of the city, the pedestrians are the intermediaries permitting a physical path of meanings linking the core of the city to its fringe, past to present and identities to cultures.

## Conclusion

In this article we have discussed the lived experience of city spaces via the lens of the theory of urban palimpsest. This analysis revealed new facets of the relationship between the urban structure, morphology, and the everyday walking experience of pedestrians and the layers and meanings that have been added to it over the years. We chose the case study of Whitechapel Rd to presents a telling example of a physical complex that has a long tradition of hosting different activities that were needed by London but which for various reasons always existed on the fringes of mainstream society. The analysis highlighted the impact of the socio-spatial roles of urban palimpsest on individuals’ everyday understanding of the changing face of Whitechapel. Along Whitechapel Rd, different urban palimpsests were identified that are anchored in ancient historical value as well as in local recognition, and much newer gathering points whose scale and parcelling have significantly changed this setting. Using an isovist technique, we showed that each key point has its own qualities and differ in terms of strength and weakness, that are influenced by location, vistas, numbers of users and functions.

An exploration of the qualities of the points and the ties between these qualities and those of the agents in them revealed two hidden socio-spatial mechanisms: the area available for actual walking trajectories at each gathering point with respect to the overall visually accessible space, and the visually accessible zones and their resultant physical organisation. As suggested by Corboz [61], it was argued that these experiences have three-dimensional depth that affect the ways people can consume and shape the public spaces. The morphological conditions of building footprints, junctions and monuments together with urban issues of centre and margin, wealth-poverty, host society and immigrants, the ancient city and its modern form all shape diverse perceptions and accessibility. While the first mechanism associates the qualities of the centre to its fringes, ancient to modern, wealth and poverty, the second mechanism enhances these relationships, reflect into them while dissolving the felt walls between them. These findings support Huyssen’s [62] claim that the components of urban palimpsest are constituted bythe spatial interactions between the physical aspects and their users. Unaware of their profound involvement in the ongoing process of the city’s formation, agents add layers and reassembled interpretations while supporting the endless process of superimposition of spatiotemporal knowledge.

From the perspective of the pedestrian, walking in different directions along the axis (East to West vs. West to East) provides a representation of Whitechapel that is alive to its contradictions. These morphological findings are consistent with the in-depth interviews that explain how the cumulative linear views towards the East and the in-depth experience of the neighbourhood provided solely by walkability offers radically different experiences. This represents the multidimensional and ever-changing face of London as a world city for different users and raises questions as to the socio-economic mechanisms that produced and continue to maintain this Janus face. A specific type of relationship between individuals and their public space is required for constructing as well as maintaining the Janus’s face experience. Our study revealed that although the urban fabric may look like a patchwork of economic activity, class relations and a cultural-ethno-racial mosaic, the vast and inclusive socio-spatial system producing this spatial arrangement operates on the basis of precise practices and local codes. There is a clear inner identity-based order, which users recognise, adjust to and obey.

These findings indicate the ’invisible hands’ [63] of the urban palimpsest on the local development. The border between the City of London and the London Borough of Tower Hamlets (Fig 2, P2) creates a property line between these two boroughs, distinguishes between their roles in the city’s everyday life. However, our findings show that the place itself is not just a matter of interpretation; there is a define property line, but there is also some unity in possible interpretations, even if they are opposed; and certain locations outside the City of London are desirable because of their hidden, yet powerful consequences for urban development. Our findings show the capacity of a given urban form for adaptation—it creates a new public sphere, embedded with contrasts and tensions, fragile but highly flexible. A Janus-faced sphere, that from one hand provides traditional roles, and from other a fertile environment for social and economic prosperity.

This Janus face interpretation in Whitechapel hints at the strong influence of urban palimpsests on patterns of public spaces. The Whitechapel axis and its interpretations shows that its local palimpsests can be an anchor for the whole city. It can tell a larger story, about the beginnings of human settlement through dichotomies of sites through time and space. The local council would like to draw more people eastwards from the City/Brick Lane/Whitechapel Gallery that they tend to visit and then leave. Urban planning can link the Boar’s Head playhouse at Aldgate (1598 to around 1616) to the Red Lion site, where a middle iron age settlement was recently excavated and will eventually be a public attraction. Since palimpsests can only be identified through walking, the ties between the two playhouses and other contemporary sites along Whitechapel Road could be exploited as part of a theatre heritage cultural trail that would accrue other benefits. As such patterns are likely to emerge within other historical cities, a more in-depth investigation could reveal the typical footprint of urban palimpsests and its consequences for urban development. As palimpsests have the power to affect the local area as well as the entire urban matrix, further comparisons between the nature of the urban layers, their visibility and urban roles may reveal the extent to which the urban palimpsest is a more common generator.
